# Effects of Vitamin D Supplementation on Inflammation, Colonic Cell Kinetics, and Microbiota in Colitis: A Review

**DOI:** 10.3390/molecules25102300

**Published:** 2020-05-14

**Authors:** Patricia Mae Garcia, Jeff Moore, David Kahan, Mee Young Hong

**Affiliations:** School of Exercise and Nutritional Sciences, San Diego State University, San Diego, CA 92182, USA; patricia.mae.garcia@gmail.com (P.M.G.); jmoore714@gmail.com (J.M.); dkahan@sdsu.edu (D.K.)

**Keywords:** vitamin D, inflammation, inflammatory bowel disease, cell kinetics, gut microbiome

## Abstract

Vitamin D is widely known to regulate bone health, but there is increasing evidence that it may also ameliorate colitis through inflammation, cell proliferation and apoptosis, and the microbiota. The purpose of this review is to systematically examine the mechanisms by which vitamin D reduces colitis. PubMed and Web of Science were searched for articles published between 2008 and 2019 using key words such as “vitamin D,” “colitis,” “inflammatory bowel disease,” “inflammation,” “apoptosis,” “cell proliferation,” and “gut bacteria”. Retrieved articles were further narrowed and it was determined whether their title and abstracts contained terminology pertaining to vitamin D in relation to colitis in human clinical trials, animal studies, and cell culture/biopsy studies, as well as selecting the best match sorting option in relation to the research question. In total, 30 studies met the established criteria. Studies consistently reported results showing that vitamin D supplementation can downregulate inflammatory pathways of COX-2, TNF-α, NF-κB, and MAPK, modify cell kinetics, and alter gut microbiome, all of which contribute to an improved state of colitis. Although vitamin D and vitamin D analogs have demonstrated positive effects against colitis, more randomized, controlled human clinical trials are needed to determine the value of vitamin D as a therapeutic agent in the treatment of colitis.

## 1. Introduction

Vitamin D is a fat-soluble vitamin deemed essential for human health. Besides being known for maintaining bone homeostasis, Vitamin D has been implicated in innate and adaptive immunity, as well as having anti-inflammatory properties [1,2]. The Dietary Reference Intakes (DRIs) for vitamin D depend on age: 400 IU vitamin D/day for children <1 year old, 600 IU vitamin D/day for people ages 1–70 years old, and 800 IU vitamin D/day for people >70 years old [2,3]. Vitamin D is obtained through sunlight, dietary supplements, and vitamin D-rich foods such as cod liver oil, tuna, sardines, milk, eggs, certain mushrooms and fortified orange juice and dairy products [2]. Supplementation with vitamin D_3_ (5000 IU) improves serum concentrations of 25-hydroxy vitamin D (25-OHD), the precursor to the physiologically-active metabolite 1,25-duhydroxyvitamin D (1,25-OHD), in deficient (<30 ng/mL) individuals [4].

Colitis is a chronic, uncontrolled inflammation of the colon and destruction of the colonic crypt affecting the gastrointestinal tract; it causes malabsorption and diarrhea, and increased risk of developing colorectal adenocarcinoma [5,6,7,8,9]. In 2015, an estimated 3.1 million adults in the United States were diagnosed with either ulcerative colitis (UC) or Crohn’s disease; most were ≥45 years old, and non-Hispanic white (estimated 345,000 people) or Hispanic (estimated 395,000 people) [10]. According to the American Cancer Society, colorectal cancer is the third most common cancer diagnosed in the United States: 1 in 22 men, and 1 in 24 women [11]. History of UC for ≥8 years is one of the major risk factors for developing colorectal cancer [11,12]. Genetic, immune, environmental, and microbial factors may also play a role in the development of colitis [9,13,14].

Vitamin D status is negatively associated with inflammatory bowel disease (IBD) activity and clinical outcomes. A systematic review and meta-analysis of 27 studies found low vitamin D status, measured via 25-OHD, to be associated with increased disease activity, mucosal inflammation, and future clinical relapse, as well as decreased quality of life among IBD patients [15]. In subgroup analyses, these associations remained significant for patients with Crohn’s disease, but only associations for disease activity and future clinical relapse remained significant among patients with ulcerative colitis [15].

Many studies have demonstrated that vitamin D improves colitis in human and animal models. In two animal studies, vitamin D deficiency contributed to increased severity and mortality in experimental colitis and even in experimental colon cancer [7,8]. Mice fed increased dietary vitamin D had significantly lower pro-inflammatory cytokines associated with colon tumor growth [8]. It has been shown that proinflammatory cytokines, such as interleukin-6 (IL-6) and tumor necrosis alpha (TNF-α), inhibit vitamin D metabolism in colon cancer cells [16]. The one-time administration of 300,000 IU vitamin D_3_ reduced inflammation in UC subjects 90 days after intervention [17]. Associations between low serum vitamin D and the incidence of UC have been posited, but the exact mechanisms have not been well defined [7,9,17]. This review paper explores potential underlying mechanisms for the effects of vitamin D on colitis in terms of inflammation, colon cell kinetics, and the microbiota.

## 2. Results

### 2.1. Dose-Dependent Effects of Vitamin D Supplementation on Ulcerative Colitis and Crohn’s Disease

Supplementation of Vitamin D_2_ (ergocalciferol) and Vitamin D_3_ (cholecalciferol) improves serum vitamin D levels, with the latter having both higher potency and efficacy [18]. The optimal dose for Vitamin D supplementation in UC patients remains to be elucidated.

Vitamin D_3_ supplementation for 90 days at 4000 IU/day outperformed supplementation at 2000 IU/day in a prospective, double-blind, randomized trial of adults with hypovitaminosis D and UC [9]. Serum Vitamin D levels rose in both groups but to a larger degree in those receiving 4000 IU/day (16.8 ng/dL) than in those receiving 2000 IU/day (5.00 ng/dL). In the 4000 IU/day group, four of the ten patients achieved normal Vitamin D levels (>30 ng/mL) following supplementation compared to only one of the eight patients in the 2000 IU/day group. Quality of life measured via the Short IBD Questionnaire (SIBDQ) increased after 90 days to a statistically significant degree in the 4000 IU/day group, but not in the 2000 IU/day group [9]. 

In another double blind, randomized clinical trial, adults with hypovitaminosis D and mild to moderate UC were supplemented with either 1000 IU/day or 2000 IU/day of Vitamin D for 12 weeks. Measures included serum 25-hydroxy vitamin D (25-OHD), inflammatory bowel disease questionnaire-9 (IBDQ-9) scores, and a simple clinical colitis activity index questionnaire (SCCAI) scores. At the end of the study, supplementing with 2000 IU/day compared to 1000 IU/day resulted in a larger mean change in 25-OHD (6.7 ng/mL vs 0.2 ng/mL), larger improvement in mean IBDQ-9 score, and larger improvement in mean SCCAI scores [19]. 

A double blind, randomized, controlled trial compared the efficacy of two doses of weekly Vitamin D_3_ supplementation, 5000 IU and 10,000 IU/10 kg bodyweight/week for six weeks in pediatric IBD patients (N = 32). Serum 25-OHD was significantly higher compared to baseline in both groups at week 8 (24.0 to 41.5 ng/mL and 23.7 to 49.2 ng/mL, respectively), but only in the higher dose group at week 12 (30.8 ng/mL, *p* < 0.132 and 35.1 ng/mL, *p* < 0.001 respectively). No patient in either group experienced hypercalcemia or serious adverse events [20].

### 2.2. Vitamin D and Inflammation

#### 2.2.1. Cyclooxygenase-2

Vitamin D has been shown to reduce inflammation and attenuate autoimmune disease by affecting both adaptive and innate immunity [21,22]. The anti-inflammatory properties of Vitamin D may work in part through the inhibition of the prostaglandin (PG) pathway. This inhibition is mediated by inhibiting the expression of cyclooxygenase-2 (COX-2), the enzyme responsible for synthesizing PGs, promoting the expression of 15-prostaglandin dehydrogenase (15-PGDH), the enzyme responsible for deactivating PGs, and disrupting PG signaling by decreasing EP and FP PG receptor expression [16,23,24] (Table 1). A human biopsy study examining COX-2 immunostaining among subjects with long-standing colitis revealed that over 90% of the biopsies from dysplastic lesions were positive for COX-2 expression [25]. The overexpression of COX-2 may accelerate tumor growth and the development of colitis to colorectal cancer through the induction of pro-oncogenic colonic crypt mutations [24,26]. Treatment of an adenocarcinoma colon cancer cell line, COGA-1A, with 10 nM 1,25-dihydroxyvitamin D (1,25-OHD) inhibited the upregulation of COX-2 mediated by TNF-α by 37% after 12 h [16]. Notably, 1,25-OHD has been found to inhibit COX-2 expression in a dose-dependent manner in murine macrophages through upregulation of thioesterase superfamily member 4 (THEM4) [27]. 

A Vitamin D analog, Ro26-2198, was found to inhibit COX-2 signaling and delay the onset of clinical colitis in an azoxymethane, dextran sulfate sodium mouse model, as well as to inhibit COX-2 induction and decrease proliferation in HCA-7 colon cancer cells. Ro26-2198 also inhibited increases in phosphoactive extracellular signal related kinase (ERK) and c-Myc intrinsic to the model [28]. 

#### 2.2.2. CYP24A1 and CYP27B1

Activation of Vitamin D in the body is regulated by two genes, CYP27B1 and CYP24A1, with the former being responsible for activation and the latter for deactivation. Regulation of these enzymes is found to be important for colitis-associated colorectal cancer prevention by ensuring appropriate serum vitamin D to maintain normal cellular growth and differentiation [39]. The expression of these genes has been shown to be altered in the inflamed colonic tissue of IBD patients, resulting in a reduction of 25-OHD and impaired Vitamin D status [40]. Unsurprisingly, altered expression and polymorphisms of these genes, as well as low serum levels of 25-OHD, are associated with colorectal cancer [39,41].

A vitamin D-deficient, CYP27B1 knockout mouse model was shown to have shorter colon length, decreased body weight, thinner intestinal mucosa, and increased colonic crypt damage than wild-type counterparts [29]. The overexpression of CYP24A1 may play a role in the low serum 25-OHD levels seen in IBD patients, and modulating its expression may allow for better regulation of serum levels. In contrast, vitamin D analog BXL-62 showed significant reduction in proinflammatory cytokine expression such as TNF-α, and induced CYP24A1 expression in peripheral blood mononuclear cells in DSS-induced colitis in mice [30]. In the first known DSS-induced colitis mice study using gene therapy, CD11b^+^/Gr1^+^ monocytes were used as the cell vehicle as well as the macrophage-specific promoter (Mac1) to deliver and upregulate CYP27B1 expression in the colon to treat colitis [31]. It has been suggested that Gr1^+^ migrates to inflamed tissues first; however, Mac1 was only highly expressed in activated macrophages [31]. These activated macrophages were engineered to express the 1α-hydroxylase gene under the promoter of the Mac1 gene, so that CYP27B1 could be transported directly to the inflamed colon [31]. Mice injected with this treatment were found to have better survival, longer colon lengths, significantly reduced proinflammatory cytokines, and even mucosal regeneration compared to controls [31]. The upregulation of CYP24A1 following Vitamin D supplementation may be disadvantageous and should be considered when treating colitis [42]. 

Some studies have found that high CYP24A1 is prevalent in cancerous lesions in the colon compared to a normal colon. In a human biopsy study, CYP24A1 expression was 20 to 35 times higher and CYP27B1 was 4 times higher among low- and high-grade cancerous lesions in the colon compared to the normal colon [24]. Unbalanced, high basal levels of CYP24A1, however, may promote the progression of tumor growth, even in the colon [39]. How high the CYP24A1 expression has to be before tumorigenesis occurs remains unknown. Feedback mechanisms may be involved, but they are not well-defined at this time.

Single nucleotide polymorphisms (SNPs) are genetic variations that may potentially become biomarkers to predict risk of certain disease states [32]. Some studies explored CYP24A1 polymorphisms and risk for UC and colorectal cancer. One such study explored the association between CYP24A1 polymorphisms and risk of colonic polyps, colon cancer, and colitis in 144 subjects with colonic polyps, 96 subjects with colon cancer, and 44 subjects with UC [31]. SNPs identified and genotyped in CYP24A1 via an assay were rs4809957, rs6068816, rs6091822, and rs8124792 [36]. It was revealed that only SNPs rs4809957 and rs6068816 were significantly associated with risk of colonic polyps, colon cancer, and UC [31]. Similar studies also explored CYP24A1 polymorphisms for colorectal cancer risk, but risks for colitis were not discussed [43,44]. More research into the role of various Vitamin D activation and deactivation gene polymorphisms in UC treatment is warranted.

#### 2.2.3. Tumor Necrosis Factor Alpha

TNF-α is a proinflammatory marker mediated by T-cells. It is one of the major pathogenic cytokines involved in the development of inflammatory bowel diseases, with elevated levels found in both CD and UC [16,45]. It has been shown that TNF-α promotes dysregulation of tight junction structures with remission following anti-TNF-α therapy [45,46,47]. If TNF-α-initiated inflammation is left unresolved, it may lead to colon cancer through angiogenesis and cell invasion [47,48]. Vitamin D may interfere with immune cells and suppress inflammatory cytokines, such as TNF-α, impeding intestinal inflammation [49].

A vitamin D analog, KH 1060, was shown to significantly increase serum vitamin D levels and decrease TNF-α levels among inflammatory bowel disease (IBD) patients. This analogue worked synergistically when combined with anti-TNF-α, resulting in lower TNF-α than either treatment alone [33]. Mice treated with vitamin D in a DSS-induced colitis model showed significantly reduced TNF-α expression compared to the control group. It was speculated that this reduction was due to a direct suppressive effect on cytokine expression or the maintenance of epithelial barrier function, thus averting innate immune system activation [34]. Similar results were seen in two other studies examining the effects of Vitamin D analogs, ZK191784 and TX527, on TNF-α expression in DSS-induced colitis murine models [35,36]. 

#### 2.2.4. Nuclear Factor Kappa B 

NF-κB is a key transcription factor that is crucial in various immune, physiological, and pathological responses; it is a major mediator of intestinal inflammation [50,51,52,53,54]. Among IBD patients, the expression of many inflammatory cytokines, such as NF-κB, IL-1 and IL-6, is increased in the intestinal mucosa [55]. In a bacterial-driven colitis and colon cancer murine model, the effects of two different Vitamin D doses were assessed: a maintenance dose of 1 IU/g of food and a high dose of 5 IU/g of food [8]. At 1 week postinfection, mice on the high-dose diet had decreased NF-κB activation in colonic epithelial cells. At 16 weeks, 41% of the mice on the maintenance-dose diet had cancer compared to only 11% of the mice on the high-dose diet. Decreased MAPK activation in the lamina propia leukocytes, inflammatory cell infiltrates, expression of proinflammatory cytokines, and IBD scores were also noted in the high-dose diet group [8]. 

Suppression of the NF-κB pathway and subsequent downstream expression of pro-inflammatory cytokines with 25-OHD has previously been confirmed in vitro [37]. A study on colon cancer-derived cells, HCT116, Caco-2, and SW480, treated with 20 nM 1,25-OHD for 24 h and followed by 100 ng/mL TNF-α for 12 h, revealed that vitamin D may maintain the intestinal epithelial barrier permeability by suppressing NF-κB activation [38].

#### 2.2.5. Mitogen Activated Protein Kinase

The mitogen-activated protein kinase (MAPK) is a family of kinases that convert a variety of signals from the cell membrane to the nucleus in response to stimuli [56]. MAPK consists of three subunits, namely the extracellular signal-regulated kinase (ERK), c-Jun NH2-terminal kinase (JNK), as well as the p38 MAPK [56]. ERK is activated in response to growth stimuli, whereas JNK and p38 MAPK are activated in response to many cellular stresses such as inflammatory cytokines and DNA damage [56,57]. A subunit of p38 MAPK, p38α, was shown to regulate cellular response to stress and play a role in maintaining intestinal tissue homeostasis by cell differentiation [58,59]. Mice with downregulated p38α in intestinal epithelial cells in an AOM/DSS-colitis study were found to develop 29 low-grade tumors and 13 high-grade colonic tumors compared to 18 low-grade tumors and 7 high-grade tumors in wild-type mice [58]. Mice on 5 IU/g vitamin D showed a 2-fold decrease of JNK activation and 7-fold decrease of p38 activation in the colon compared to controls, suggesting that vitamin D may be used to treat IBD early on and prevent progression to colon cancer [8].

#### 2.2.6. Retinoid X Receptor Alpha

RXRα belongs to the steroid nuclear receptor super family and is downregulated in murine colitis, colorectal cancer, and colon cancer cell lines, although the exact mechanism is yet to be identified [13,60]. RXRα^+/−^ heterozygous mice treated with AOM/DSS showed significantly reduced RXRα expression and had a shorter colon length of 57.6 ± 9.4 mm compared to 67.8 ± 6.1 mm in the control group [60,61]. In the same study, heterozygous mice increased Snail1 and Snail2 expressions, which are transcription factors upregulated by inflammatory cytokines [60,61].

RXRα is also involved in the vitamin D signaling pathway via heterodimerization with VDR [60,62,63]. The biological activity of vitamin D may influence the immune system and inflammation by inducing VDR expression in the colon [6,9,14,64]. The VDR may play an anti-inflammatory role, especially in people with UC, as reduced VDR increases mucosal inflammation and barrier dysfunction [14]. In an experimental colitis mouse model, mice fed a vitamin D-deficient diet showed a lower serum vitamin D level of 20.1 ± 5.1 ng/mL, shortened colon length of 44.1 ± 4.6 mm, higher mortality rate at 7–10%, and lower VDR and RXRα expressions compared to control mice with a serum vitamin D level of 36.5 ± 4.7 ng/mL and colon length of 63.3 ± 3.8 mm [13].

Together with vitamin D and its proposed anti-inflammatory properties, vitamin D may reduce colitis by regulating COX-2, TNF-α, NF-κB through IkBa expression, RXR signaling, and vitamin D-activating enzymes CYP24A1 and CYP27B1.

### 2.3. Vitamin D and Cell Kinetics 

#### 2.3.1. Cell Proliferation

Normal colonic mucosa are made up of epithelial cells with folds called crypts, which are consistent throughout the colon [65,66]. Crypt renewal occurs at the bottom of the crypts where intestinal stem cells generate cell proliferation and differentiation, and thus replenish the epithelium [65,66,67]. In colitis, however, inflammation ultimately causes crypt disruption and architectural irregularities [65,66]. Vitamin D has been shown to promote mucosal regeneration through the modulation of signaling pathways, including VDR [65] (Table 2). Treatment of 10^−8^ M 1,25-OHD (calcitriol) in a cell study for 24 hr revealed increased expression of tight junction proteins, suggesting that vitamin D may play a role in maintaining the integrity of the tight junction complex [65]. VDR knockout mice showed impaired mucosal wound healing after DSS treatment, to the point where severe ulceration caused total crypt loss compared to wild-type mice [65].

Ki-67 is a nuclear protein constitutively expressed in mammalian cells, and is widely used as a cell proliferation marker in colitis and colon cancer [68]. DSS-treated rats had a significantly increased Ki-67 proliferation index compared to mice without DSS treatment [67]. Mice treated with vitamin D_3_ at 15, 30 and 60 IU⋅g^−1^⋅w^−1^ had significantly inhibited colonic cell proliferation through Ki-67 staining by 17%, 35%, and 41%, respectively, in an AOM/DSS-treated colitis model compared to controls [69]. Patients with colitis have significantly increased Ki-67 expression in low-grade dysplasia, high-grade dysplasia, and carcinoma compared to normal epithelium [70]. A longstanding UC biopsy study also revealed elevated Ki-67 expression in both low- and high-grade dysplasia [25].

Another biopsy study showed an inverse relationship between low serum vitamin D and Ki-67 expression among patients with precancerous and cancerous colon cells, as high proliferation is associated with cell malignancy [71]. Patients who had ≥50% Ki-67 expression in colon cells had a mean serum vitamin D level of 6.27 ng/mL; patients who had ≤50% Ki-67 expression had a mean serum vitamin D level of 13.42 ng/mL; and patients who had <15% Ki-67 expression and a mean serum vitamin D level of 20.53 ng/mL [71]. Treatment of 800 IU vitamin D/day for six months increased p21 expression by 242% compared to placebo [72]. A cyclin-dependent kinase inhibitor, p21 plays a critical role in cell differentiation, cell cycle control, apoptosis modulation, and tumorigenesis [72]. Vitamin D reduces colitis by promoting colonic mucosa regeneration and regulating cell proliferation via modulation of p21 expression.

**Table 2:** Characteristics of Published Clinical Trials Examining Vitamin D and Cell Kinetics.

Author (Year)	Study Type, Population	Measurements	Results	Conclusion
Kong et al. (2008) [65]	VDR^+/+^ (wild-type) and VDR^−/−^ (KO) mice 2.5% DSS treatment; cell study	Colonic samples; histology, immunostaining, electron microscopy, Western blotting, RT-PCR	VDR KO mice had more severe colitis/ulceration, symptoms like diarrhea, gross bleeding, rectal prolapse, ↑ mucosal permeability, impaired epithelial junction compared to wild-type mice; vitamin D treatment ↑ tight junction proteins in 24 h	Vitamin D may protect intestinal epithelial barrier by increasing tight junction proteins
Xin et al. (2017) [69]	C57BL/6 mice 12.5 mg/kg AOM, 2.5% DSS treatment; Cell culture (SW480 cells) w/ 100 nmol/L 1,25-OHD treatment	Serum vitamin D, calcium, creatinine levels; vitamin D (15, 30, or 60 IU⋅g^−1^⋅w^−1^); transient transfection, luciferase assay; RNA isolation, RT-PCR, immunoprecipitation and Western blotting	AOM/DSS treatment ↓ serum vitamin D levels; vitamin D administration significantly ↓ the number and colorectal neoplasms in a dose-dependent manner by up to 59%, ameliorated colon length shortening, ↓ cell proliferation in the colon ↓ β-catenin expression in vivo, ↑β-catenin binding to E-cadherin *in vivo*	Vitamin D is beneficial against AOM-DSS-induced colitis-associated carcinogenesis E-cadherin upregulation is beneficial on vitamin D’s preventive effect on β-catenin
Fedirko et al. (2009) [72]	Randomized, double-blind, placebo-controlled, clinical trial in men and women (N = 92) with confirmed colorectal adenoma	Automated immunohistochemistry and image analysis to detect and quantify expression and distribution of p21^waf1/cip1^, MIB-1, and hTERT in colorectal crypts	p21 expression ↑ 2-fold in Vitamin D and Vitamin D plus calcium but not in placebo group; no change in MIB-1 or hTERT expression; proportion of hTERT extending into upper 40% of the crypts ↓ by 15%	Calcium and Vitamin D promote differentiation of colorectal epithelial cells and may regulate colorectal crypt proliferative zone in patients with sporadic adenoma
Zhu et al. (2015) [73]	Biopsies from IBD patients; C57BL6/J mice treated with 100 mg/kg TNBS and randomly divided into 2 groups: TNBS and control w/ either paricalcitol 0.5 µg/kg body weight or dissolvent only	Western blotting, hematoxylin/eosin staining, serum vitamin D levels, FTC-dextran intestinal permeability	↓ serum vitamin D and VDR among IBD patients; paricalcitol ↓ TNBS-induced colitis (i.e., minor weight loss, mild colitis, minimal histological damage), protects intestinal permeability, inhibits intestinal epithelial cell apoptosis by ↓ PUMA expression	Vitamin D and vitamin D analogs show promise when treating IBD by downregulating PUMA expression

#### 2.3.2. E-Cadherin 

E-cadherin is a transmembrane linker protein and calcium-dependent cell adhesion molecule for colon crypt structure and function, making it important for mediating and maintaining intestinal homeostasis and epithelial architecture [74]. The loss of E-cadherin expression transitions adenoma to carcinoma, is a hallmark of colon carcinogenesis, and is prevalent in colitis [75,76]. The deletion of E-cadherin in mice resulted in severe loss of body weight, severe loss of intestinal epithelial structure, an increased number of apoptotic cells, impaired bacterial defense, and length reduction of the small intestine compared to controls [76]. In an AOM/DSS-colitis mice study, vitamin D_3_ supplementation (15, 30, and 60 IU·g^−1^·w^−1^ 3x/week) showed significant preventive effects in high doses, decreased the number of neoplasms by 33–63%, and increased E-cadherin expression, suggesting inhibition of colonic carcinogenesis progression [69]. E-cadherin in epithelial and immune cells was found to be expressed less in CYP27B1 knockout mice compared to their wild type counterparts in this DSS-induced colitis study [77]. These studies suggest that vitamin D improves colitis and prevents colon cancer progression by enhancing E-cadherin expression.

#### 2.3.3. Wnt/β-Catenin 

Wnt signaling is crucially involved in stem cell maintenance and epithelial cell proliferation [78,79,80]. Wnt is activated in most colon cancers; therefore, it can be used as a potential biomarker for colon health [78,81,82]. Wnt may also contribute to carcinogenesis from colitis to colon cancer [78,81,82]. Wnt is capable of signaling in a paracrine and autocrine manner, and the absence of Wnt promotes the degradation of β-catenin [79]. β-catenin is a key component of the Wnt pathway, and the activation of β-catenin predisposes humans and mice to both colitis and colon cancer [78,79,82,83]. In a biopsy study, 100% of the patients in the colitis-associated colorectal cancer group demonstrated β-catenin expression compared to none of the patients in the control group [81]. In another biopsy study, there were significantly more colonic Wnt and β-catenin stained cells in colitis patients (2.5-fold) and colorectal cancer patients (4.5-fold increase) compared to colon tissue from healthy controls [82]. Tumor samples extracted from patients with chronic UC and colon cancer were found to have elevated β-catenin expression after staining compared to noncancerous colonic tissues [83].

Supplementation of vitamin D_3_ (15, 30, and 60 IU·g^−1^·w^−1^ 3x/week) in an AOM/DSS-colitis mouse model revealed decreased β-catenin, number and burden of colorectal neoplasms, and shortening of colon length, all in a dose-dependent manner [69]. Many other studies have explored the effects of vitamin D on the Wnt signaling pathway in the colon, but few have done so specifically in colitis [84,85].

#### 2.3.4. Cell Apoptosis 

Apoptosis is a process of natural cell death by eliminating genetically-mutated cells [86,87,88]. Homeostasis of the colonic epithelium requires a balance among cell proliferation, necrosis, and apoptosis [86,87,88,89]. Excessive intestinal epithelial cell apoptosis is a major cause of loss of epithelial cells and mucosal permeability, contributing to the development of colitis [7,90,91]. In a DSS-induced colitis rat model, the epithelial apoptotic index was significantly increased, at 7.2% ± 1.2% compared to 2.8% ± 1.2% in the control group [90]. Increased apoptosis in the DSS-treated colitis model showed a significantly decreased number of intact colonic crypts [67]

VDR signaling has been shown to prevent apoptosis by downregulating p53-upregulated modulator of apoptosis (PUMA) by blocking NF-κB in TNBS-induced colitis [91]. A tumor suppressing gene, p53 is crucial for cell fate and prevents the spread of damaged cells [91]. Along with p53, PUMA is a potent inducer of apoptosis that is correlated with the severity of colitis, and is crucial to mediating intestinal epithelial cell apoptosis [7,92]. In an experimental colitis mouse model, VDR knockout mice developed more severe colitis with significant colonic epithelial cell apoptosis compared to control mice [91]. In another study, VDR knockout mice had significantly increased apoptotic epithelial cells compared to wild-type mice [7]. Mice treated with a vitamin D analog, paricalcitol, in a 2,4,6-trinitrobenzene sulfonic acid (TNBS) induced colitis model, had decreased severity of colitis compared to controls mediated in part through an inhibition of intestinal epithelial cell (IEC) apoptosis from a downregulation of PUMA expression [73]. The absence of PUMA was found to relieve DSS- and TNBS-induced colitis, as well as to inhibit IEC apoptosis in PUMA-knockout mice compared to wildtype mice [92]. Human biopsies have revealed elevated levels of p53 and PUMA in the inflamed mucosal tissues of UC patients compared to those of healthy controls [93]. Vitamin D protects against colitis through the prevention of apoptosis via downregulation of PUMA.

### 2.4. Vitamin D and Microbiota

The gastrointestinal tract houses a variety of microbiota that are important for nutrient absorption, immunity, and intestinal epithelial barrier homeostasis [94,95]. Dysbiosis or abnormal interactions between the host and the gut microbiota lead to gastrointestinal diseases such as colitis [94,95,96,97]. UC patients have an unstable microbiota profile and reduced bacterial species richness compared to healthy patients [98]. Probiotics aid in intestinal homeostasis, and studies have shown potential therapeutic benefits of probiotics in colitis [94,95,96]. Probiotics have been shown to enhance VDR transcriptional activity, induce remission, reduce clinical activity scores, and prevent pouchitis, a complication in colitis following ileal pouch-anal anastomosis surgery [94,95,96]. In an experimental *Salmonella* colitis mouse model, probiotics *Lactobacillus rhamnosus* strain GG and *Lactobacillus plantarum* increased VDR expression and signaling, suggesting that VDR is necessary to prevent pathogenic invasion in the colon [96] (Table 3).

Serum vitamin D levels are associated with the function and composition of the intestinal microbiome [99,100]. *Clostridium difficile* (*C. difficile*) is a Gram-positive, anaerobic, spore-forming bacillus associated with diarrhea, sepsis, megacolon, and pseudomembranous colitis [101]. *C. difficile* is associated with a four-fold increase in mortality, increased hospitalization, and risk for colectomy up to 5 years after the first episode [102]. *C. difficile*-induced colitis is a costly and common healthcare-associated infection following antibiotic treatment, causing approximately 29,000 annual deaths in the United States [103]. *C. difficile* infection (CDI) is correlated with low serum vitamin D in IBD patients (mean of 20.4 ng/mL) versus non-CDI IBD patients (mean of 27.1 ng/mL), with a 4% reduction in CDI risk associated with each 1 ng/mL increase in plasma 25-OHD [102]. One innate line of defense against the toxic effects of *C. difficile* is cathelicidin, an anti-inflammatory, antimicrobial peptide activated by plasma 25-OHD [98,102,104]. Cathelicidin levels are higher in UC patients than healthy controls, but decrease with disease severity [98,102,104] A randomized, controlled trial in UC patients found a 3-fold increase in the cathelicidin precursor, human cationic antimicrobial protein 18 (hCAP18), gene expression following a single intramuscular injection containing 300,000 IU Vitamin D_3_ [17]. Serum levels of 25-OHD are positively associated with serum and colonic cathelicidin among UC patients. Serum cathelicidin is negatively associated with histologic inflammation, and clinical relapse risk though these associations did not remain significant in multivariate analyses. In these multivariate analyses, only 25-OHD levels ≤35 ng/mL were independently associated with an increase in histologic inflammation and baseline histologic inflammation independently with an increase in clinical relapse risk [105]. 

It has been suggested that VDR acts as a master regulator of intestinal inflammation by regulating both autophagy and antimicrobial peptide production [106]. VDR KO mice have a higher susceptibility to DSS-induced colitis and subsequent dysbiosis with fewer butyrate-producing bacteria and increased *E. coli* and *Bacteriocides* [106]. Decreased levels of butyrate-producing bacteria and butyrate itself have been noted in UC patients relative to healthy controls [107]. The pretreatment of human IEC with butyrate was found to increase VDR expression in a dose-dependent manner [106].

Angiogenin-4 (Ang4) is an antimicrobial protein involved in host containment of enteric bacteria [108]. The expression of Ang4 is downregulated in the colon after DSS treatment, and even lower in vitamin D-deficient mice receiving either tap water or DSS [108]. Decreased Ang4 expression in vitamin D-deficient mice leads to homeostasis disturbance in the colon compared to vitamin D sufficient mice [108]. It is possible that vitamin D may regulate enteric bacteria by promoting Ang4 expression, but the mechanisms have not yet been identified [108].

Colonic tissue samples from IBD patients had phylogenetic distribution from 4 phyla of bacteria: *Firmicutes* (49%), *Bacteroidetes* (23%), *Proteobacteria* (21%), and *Actinobacteria* (5%) [109]. A stool sample study revealed that *Firmicutes* (60%), *Verrumicrobiota* (25%), and *Bacteroidetes* (13%) were the main phyla from an IBD patient, whereas *Firmicutes* (59%), *Bacteroidetes* (28%), and *Actinobacteria* (13%) were found from a colon carcinoma patient [110]. 

The administration of vitamin D to patients with UC has been shown to reduce intestinal inflammation. These reductions coincided with increases in Enterobacteriaceae and a trend of reduction of the mucolytic species *Ruminococcus gnavus*, but not with changes in the overall fecal microbial diversity [98]. The supplementation of vitamin D in individuals with Crohn’s disease has also been shown to influence microbial communities. One week of supplementation increased the abundance of *Alistipes (Bacteroidetes)*; however, by the second week prevalence of *Bacteroidetes* decreased, while *Blautia*, *Firmicutes*, *Fusicatenibacter, Intestinibacter*, and *Veillonella* increased. By the third week *Parabacteroides* were at their highest. By week 4, *Lactobacillus* and *Megasphera* were at their highest. From week 0 to week 4 of vitamin D supplementation, the number of bacterial taxa decreased [111]. 

There are bacterial changes observed in feces between Cyp KO mice and Cyp wild-type mice [77]. Cyp KO and VDR KO mice had more *Bacteroidetes* and *Proteobacteria* phyla, but fewer *Firmicutes* and *Deferribacteres* phyla than wild-type mice [77]. However, vitamin D treatment reduced the numbers of the Helicobacteraceae family from the *Proteobacteria* phylum in Cyp KO mice [77]. Vitamin D protects against colitis through VDR by regulating the gut microbiota and decreasing pathogenic infections in the colon.

**Table 3:** Characteristics of Published Clinical Trials Examining Vitamin D and the Microbiota.

Author (Year)	Study Type, Population	Measurements	Results	Conclusion
Sharifi et al. (2016) [17]	Double-blind, randomized, controlled trial with parallel design including UC patients in remission (N = 90)	Serum levels of 25-OHD, PTH, Calcium, ESR, and hs-CRP and cathelicidin expression via qRT-PCR pre and post 90 days of 300,000 IU intramuscular Vitamin D or saline placebo	From pre to post, ↑ 25 -OH D, ↑ hCAP18 (human cationic antimicrobial protein 18) gene expression, ↓ Hs-CRP and ↓ ESR only in Vitamin D group	Supplementation of Vitamin D may benefit UC patients as evidenced by ↓ ESR, ↓ hs-CRP, and ↑ LL37 gene expression
Wu et al. (2015) [96]	C57BL/6 mice and VDR KO C57BL/6 mice; *Salmonella*-induced colitis mouse model	*Salmonella* infection Histology, Western blotting; immunofluorescence; VDR protein expression transcriptional activity; RT-PCR	VDR KO mice significantly lost more body weight, probiotics had no protection from *Salmonella*-induced colitis, and more *Salmonella* infection occurred compared to VDR^+/+^ mice; *Lactobacillus rhamnosus* strain GG (LGG) and *Lactobacillus plantarum* (LP) ↑ VDR expression in vitro	Probiotics are suggested to enhance VDR expression and may help protect against colitis
Ananthakrishnan et al. (2014) [102]	Multi-institutional cohort of IBD patients (n = 3188); 35 patients developed *Clostridium difficile* infection (CDI) w/ mean age of 60.5 years old	Serum vitamin D level measurement via radioimmunoassay, high-performance liquid chromatography w/ mass spectrometry	IBD patients with CDI who died had a mean serum average of 12.8 + 8.1 ng/mL compared to IBD patients who remained alive at the end of follow up (24.3 + 13.2 ng/mL); 1 ng/mL increase in serum vitamin D = w/ a 4% CDI reduction	Higher serum vitamin D levels were associated with a decreased CDI risk
Wu et al. (2015) [106]	Conditional VDR KO mouse model (VDR^ΔIEC^) 5% DSS treatment, butyrate treatment (fermentation product of gut microbes)	Vitamin D-responsive element transcriptional activity, Western blotting, intestine histology, immunofluorescence, lysotracker staining, RT-PCR, chromatin immunoprecipitation (CHIP) assay	VDR^ΔIEC^ susceptible to DSS-induced colitis, ↓ weight, ↓ cecum length, fecal blood present, ↑ intestinal inflammation, fewer butyrate-producing bacteria and butyrate compared to no DSS treatment; butyrate ↑ VDR expression and inhibited inflammation; ↑ *E. coli* and *Bacteriocides*	VDR may help regulate intestinal homeostasis via production of antimicrobial peptides
Lagishetty et al. (2010) [108]	C57BL/6 mice raised on a normal diet (n = 16) or vitamin D-deficient diet (n = 16) × 6 weeks 2.5% DSS treatment	Tissue collection/analysis, clinical colitis score, histological colitis score, flow cytometry, RT-PCR; Ang4 immunohistochemical analysis	Vitamin D-deficient mice treated with DSS had more severe colitis, ↓ colonic Ang4 expression, ↑ bacterial infiltration compared to mice with normal diet; DSS-treated vitamin D-deficient mice had ↓ serum vitamin D level (2.5 ± 0.1 ng/mL) compared to mice with normal diet (24.4 ± 1.8 ng/mL)	Ang4 promotes bacterial innate immunity against gut microbes and its function under a low serum vitamin D levels is impeded
Ooi et al. (2013) [76]	Cyp wild-type mice, Cyp KO mice, VDR wild-type mice, VDR KO mice 3.5% DSS treatment x 5 days	Fecal samples from mice Denaturing gradient gel electrophoresis, metagenomic analysis, RT-PCR, cell isolation	*Firmicutes* and *Bacteroidetes* are the dominant phyla in all mice; Cyp KO and VDR KO had ↓ *Firmicutes* and *Deferribacteres*, ↑ *Proteobacteria* and *Bacteroidetes* phyla than wild-type mice; vitamin D treatment ↓ *Helicobacteraceae*	Vitamin D and VDR may influence microbiome composition and protect against GI insults
Garg et al. (2018) [98]	Patients with active UC, inactive UC and noninflammatory bowel disease controls; received 40,000 units cholecalciferol weekly for 8 weeks.	Markers of inflammation and fecal microbiota	Patients with active UC ↓ faecal calprotectin levels; this did not change in patients with inactive UC or non-IBD controls. No changes in overall fecal bacterial diversity. were noted although a significant ↑ in Enterobacteriaceae abundance in patients with UC	Vitamin D supplementation reduced intestinal inflammation in patients with active UC, with an increase in Enterobacteriaceae and a trend to reduction in the mucolytic species *Ruminococcus gnavus* but no change in overall fecal microbial diversity
Schaffler et al. (2018) [111]	A prospective, longitudinal, controlled interventional analysis in seven patients with Crohn’s disease (CD) in clinical remission and 10 healthy controls (HC); orally administration of vitamin D	Intestinal bacterial composition	↓ bacterial richness in the CD microbiome. ↑ *Alistipes, Barnesiella*, unclassified Porphyromonadaceae (both Actinobacteria), *Roseburia, Anaerotruncus, Subdoligranulum* and an unclassified Ruminococaceae (all Firmicutes)	Vitamin D has a specific influence on the bacterial communities in CD, but not in HC. Administration of vitamin D may have a positive effect in CD by modulating the intestinal bacterial composition and also by increasing the abundance of potential beneficial bacterial strains. Vitamin D did not change the bacterial communities in HC.

## 3. Discussion 

Evidence from tissue biopsies, cell line studies, murine models, and clinical human trials suggest that vitamin D may be beneficial against colitis by reducing inflammation, inhibiting cell proliferation and apoptosis, and regulating the gut microbiota. Furthermore, these benefits are often exerted in a dose-dependent manner with no clearly established ceiling. The reduction in inflammation appears to be multifactorial, resulting from a modification of the expression of COX-2, CYP24A1 and CYP27B1, MAPK, and RXRα, and a reduction of cytokines including TNF-α and NF-kB. A decrease in cell proliferation and β-catenin and increase in cell apoptosis and E-cadherin expression also helps explain the beneficial effect of Vitamin D. Pathogenic alterations in the composition of gut microbiota associated with colitis may be mitigated by Vitamin D supplementation through increased VDR signaling and Ang4 expression. Most current evidence supporting the benefit of Vitamin D supplementations to colitis stems from cell line studies and murine models. While the evidence from human-based genetic studies and tissue biopsies suggest similar underlying mechanisms, the success of Vitamin D in treating colitis in murine models needs to be confirmed in human clinical trials. Furthermore, while Vitamin D analogs can be effective in certain models, evidence of their efficacy in humans is lacking. Mechanistic evidence from animal and cell models, such as that cited within this review, should be considered when developing much needed human clinical trials.

The beneficial effects of vitamin D on IBD also appear to be mediated through actions on inflammatory cytokines including TNF- α, IL-6, and IL-12 and anti-inflammatory cytokines including IL-4, IL-10, and TGB- β. The ratio of these anti-inflammatory to inflammatory cytokines is improved by restoring vitamin D status via supplementation in IBD patients who otherwise often have insufficient levels [112]. 

According to our findings, VDR plays a critical role in the action of vitamin D against colitis. Inflammation-related signaling including E-cadherin, RXRα, and wnt/β-catenin seems to work via VDR. VDR KO mice generally suffer from impaired mucosal healing, severe crypt loss, and higher apoptotic ratio. These studies indicate that VDR status is crucial and appears to be some sort of defense mechanism in colitis. Colitis decreases cell proliferation and increases apoptosis; a lack of VDR in several mice studies was shown to affect cell proliferation and apoptosis. VDR is involved in maintaining the tight junction and intestinal epithelial barriers that regulate cell proliferation. 

The role of the microbiome is very important in colitis prevention or treatment. However, more studies are needed on the potential mechanisms of vitamin D supplementation on the microbiota in IBD patients. 

Due to the short half-life and lower circulating levels of the active form, 1,25-OHD, its precursor, 25-OHD, is often used to assess the vitamin D status in the body [113]. However, in order for 25-OHD to accurately reflect 1,25-OHD levels, the 1α-hydroxylating enzyme CYP27B1, which converts the former to the latter, would need to be optimally expressed [29]. Similarly, optimal levels of the catabolizing hydroxylase CYP24A1, which degrades 1,25-OHD, would be essential. CYP24A1 is induced mainly by 1,25-OHD, and CYP24A1 reduces elevated local 1,25-OHD levels in a negative feedback manner [39]. In those with colitis, the ratio of CYP27B1 to CYP24A1 may prove to be important for successful treatment [39].

In addition, CYP27B1 polymorphisms can lead to Vitamin D insufficiency and supplementation of Vitamin D increases CYP24A1 expression. Some studies have shown that high CYP24A1 can induce colon carcinogenesis, but no current studies have indicated how high it would have to be to do so. The role of CYP27B1 and CYP24A1 in regulating serum Vitamin D levels is both complex and consequential. Future studies should include the dose effects of vitamin D on CYP24A1 expression to determine the threshold at which CYP24A1 promotes colon carcinogenesis.

It was observed that the criteria for defining Vitamin D statuses varied between studies [9,17,29,38,99]. While the Institute of Medicine has specified serum vitamin D thresholds indicative of insufficiency and deficiency with regards to bone health [114], levels which optimize human immune function remain unclear and require further investigation.

Vitamin D analogs have shown promising results, and their utility in treating colitis should continue to be explored. Administration of 1,24(OH)_2_D_2_ was found to have similar effects compared to 1,25(OH)_2_D_2_ on reducing colitis symptoms, but 1,24(OH)_2_D_2_ presents less risk in raising serum calcium levels [63]. Having high serum calcium, or hypercalcemia, is a potentially life-threatening condition that can cause progressive kidney failure, dehydration, and granulomatous diseases such as tuberculosis and sarcoidosis [106]. The relationship between calcium and vitamin D, specifically concerning the various types of vitamin D, should be further investigated as potential therapeutic options for colitis.

Animal studies remain popular, as genetic techniques can optimize disease models to better understand the pathology of ulcerative colitis. There are limited human clinical trials on the effect of vitamin D on colitis. More randomized, controlled human clinical trials are needed. In conclusion, vitamin D may benefit sufferers of colitis by reducing inflammatory cytokines, maintaining intestinal epithelial cell integrity by modulation of cell proliferation and apoptosis, and reducing pathogenic infections. 

## 4. Materials and Methods 

PubMed and Web of Science were the primary databases used to search for articles—published between 2008 and 2019—using key words such as “vitamin D and colitis,” “vitamin D and inflammatory bowel disease,” “inflammation,” “apoptosis,” “cell proliferation,” and “gut bacteria”. PubMed initially yielded 447 articles that were then evaluated further. Secondary inclusion criteria were based on whether or not titles and abstracts included “vitamin D” in relation to colitis in human subjects, animal models, and cell culture studies, as well as selecting the best match sorting option on PubMed according to key words. Upon further evaluation, 251 articles met the secondary criteria. After further checking based on the criteria of original studies and mechanism-based vitamin D intervention on colitis, 30 articles were finally chosen that were most relevant to vitamin D and colitis on inflammation (Table 1), cell kinetics (Table 2), and the microbiota (Table 3).

## Figures and Tables

**Table 1:** Characteristics of Published Clinical Trials Examining Vitamin D and Inflammation.

Author (Year)	Study Type, Population	Measurements	Results	Conclusion
Brozek et al. (2012) [24]	Factor and cluster analysis of 105 patients undergoing primary curative surgery for adenocarcinomas	Expression of *VDR*, *CYP27B1*, *CYP24A1* and *COX-2* in relation to tumor grade, anatomical location, and gender	Compared to adjacent mucosa mRNA expression in cancerous lesions was ↑ in CYP27B1 (4-fold in low/high grade cancers), *CYP24A1* (20-35-fold in low/high grade lesions) and *COX-2* (2-fold in high grade cancers) but not *VDR;* in distal colon tumors *CYP27B1* expression 2-fold ↑ in men than women	Antagonism between *COX-2* and Vitamin D could be important factor in epithelial colorectal cancer cell growth; differences in *COX-2* expression could influence variation in incidence at different anatomical subsites; cancer incidence gender-specific differences correlate with age
Frăţilă & Iliaş (2013) [25]	Retrospective study 2006–2010 80 patients w/ longstanding ulcerative colitis (LUC) dx (42 women, 38 men) 53.5 ± 14.2 years old	Colonoscopy biopsies: 20 high-grade dysplasia (HGD), 20 low grade dysplasia (LGD), 20 regenerative atypia, 20 indefinite for dysplasia; immunohistochemical (IHC) staining applied for Ki-67, clone MIB-1, and anti-COX-2	COX-2 is positive in 72.5% LUC biopsies; LGD and HGD had Ki-67 staining	IHC staining may be used to manage increased colorectal cancer risk in LUC patients
Leedham et al. (2009) [26]	Mutation burden analysis of individual crypts across colitis associated neoplasms	PCR and sequencing analysis to establish individual crypt adenomatous polyposis coli (APC), p53, K-RAS, and 17p loss of heterozygosity mutation burden	Monoclonality observed in most lesions typically from *p53* lesion and occasionally *K-RAS*	*p53* mutation was the initiator in the majority of lesions; *K-RAS* activation found to be gatekeeping mutation
Wang et al. (2014) [27]	Macrophages from COX-2 KO and COX^Neo/Neo^ mice	COX-2 expression and PG expression in the presence and absence of LPS stimulation	1,25-OHD results in dose-dependent inhibition of COX-2 expression and phosphorylation of Akt and IκBα in murine macrophages with and without LPS stimulation	Vitamin D influences inflammation and supplementation could improve chronic inflammatory diseases via targeting THEM4/Akt/NF-κB signaling
Hummel et al. (2014) [16]	Adenocarcinoma cell line COGA-1A culture and treatment	COGA-1A cells treated with 10 nM 1,25-OHD, 100 ng/mL IL-6, 50 ng/mL or combination of for 6, 12, and 24 hrs; total RNA isolation; reverse transcription polymerase chain reaction (RT-PCR)	COGA-1A cells + 1,25-OHD = ↑ VDR expression; IL-6 ↑ CYP24A1 expression 3x; COX-2 and 15-PGH expression “unresponsive” with 1,25-OHD in COGA-1A cells, but TNF-α “highly ↑ COX-2 expression”	TNF-α and IL-6 inhibited vit D expression-activating gene CYP27B1 in COGA-1A cells
Fichera et al. (2007) [28]	Male A/J mice (25 g) AOM/DSS-induced colitis	Vitamin D analog Ro26-2198 (0.01 µg/kg body wt/day x 28 days); severity of colitis assessed via the Disease Activity Index; hematoxylin and eosin colonic sections examined for dysplasia; colonic lysates assessed for c-Myc, COX-2, phosphor-(active) extracellular signal regulated kinase (ERK) via Western blotting	DSS treatment ↑ c-Myc 15-fold, ERK 10-fold, COX-2 2.5-fold Ro26-2198 ↓ proliferative (c-Myc, ERK) and pro-inflammatory (COX-2) signals and dysplasia progression	Vitamin D analog can be considered when treating colitis
Liu et al. (2016) [29]	25-OHD 1α-hydroxylase knockout (Cyp27b1^−/−^) mice fed high calcium, phosphate, and lactose rescue diet	Body weight, colon length, and colonic histologic structure	Cyp27b1^-/-^ had ↓ bodyweight, colon length, colon length to bodyweight ratio, mucosa thickness and ↑ crypt damage	1,25-OHD may influence colon inflammation and cancer development and progression
Laverny et al. (2010) [30]	Peripheral blood mononuclear cells (PBMC) obtained from IBD patients (21 ulcerative colitis patients, 22 Crohn’s Disease patients) 26 males, 13 females; C57BL/6 mice w/ 3% DSS treatment; cell study	VDR agonist BXL-62 administration; histology; cytokine quantification; RT-PCR	BXL-62 has 3x less calcemic activity, ↓ proinflammatory cytokines in cells of IBD patients, ↑ CYP24A1 expression, ameliorates experimental colitis compared to 1,25-OHD	BXL-62 is a VDR agonist that does not promote hypercalcemia, ↓ in vitro pro-inflammatory cytokines, and may be used as IBD treatment
Li et al. (2015) [31]	Gene therapy (regulate CYP27B1 expression via CD11b^+^/Gr1^+^ monocytes) in murine DSS-induced IBD model	Survival rate, weight, colonic structure, mucosal regeneration index, cytokine expression	↑ survival, body weight gain, colon length, mucosal regeneration and ↓ proinflammatory cytokines (TNF-α, IL-1β, IL-6, IL-12, IL-23, Th1 and Th17)	This preliminary evidence of a monocyte-based adoptive CYP27B1 gene therapy in a murine IBD model may lead to novel therapy for autoimmune disease like IBD
Chen et al. (2017) [32]	Genotyping of patients with colonic polyps (n = 144), colon cancer (n = 96), and UC (n = 44) to determine correlations between CYP24A1 SNPs and diseases	Genotyping of four SNPs (rs4809957, rs6068816, rs6091822 and rs8124792) and their association with colonic polyps, colon cancer, and UC	*CYP24A1* polymorphisms rs4809957 A/G and rs6068816 C/T were associated with colonic polyps, colon cancer, and UC	rs4809957, rs6068816, rs6091822 and rs8124792 are related to risk of colonic polyps and cancer; the A allele in rs8124792 may indicate colonic polyps and cancer not UC
Stio et al. (2006) [33]	PBMC of IBD patients; KH 1060 (vitamin D analog) treatment	[^3^H]thymidine incorporation; ELISA kit; VDR levels w/ Western blotting	KH 1060 ↓ PBMC proliferation, ↓ TNF-α, ↑ VDR expression	KH 1060 can be used on IBD patients to ↓ TNF-α
Zhao et al. (2012) [34]	C57BL/6 mice (n = 30), equally divided among 3 groups: 2% DSS treatment, control, and vitamin D	Serum vitamin D levels, Rachmilewitz DAI (disease activity), colonic injury/inflammation; myeloperoxidase (MPO) activity, mesenteric lymph nodes cells (MLNs) and LPMC isolation; immunohistochemistry; immunofluorescence; *in vivo*/*in* vitro permeability; TEER measurement; qT-PCR; Western blotting	Serum vitamin D levels were ↓ in DSS group compared to control; DSS damaged mucosal barrier and ↑ colonic inflammation, ↑ weight loss; vitamin D inhibited colonic inflammation, maintained intestinal permeability, preserved colonic structural integrity, prevented DSS-induced tight junction disruption	Vitamin D warrants therapeutic potential against IBD
Strauch et al. (2007) [35]	Female Balb/c mice w/ 3% DSS treatment; ZK191784 (vitamin D analog) 100 μg/kg per day	Histological examination; ELISA of MLNs; RT-PCR; immunohistochemistry	ZK191784 treatment ameliorates acute and chronic DSS-induced colitis, suppresses proinflammatory cytokine secretion by MLNs and primary dendritic cells	Another study by which vitamin D analog is shown to be beneficial against experimental colitis
Verlinden et al. (2013) [36]	DSS-induced colitis in mice; TX527 (vitamin D analog)	Histological examination, transcript levels of proinflammatory cytokines	TX527 ameliorated DSS-colitis symptoms by ↓ diarrhea and bleeding, ↓ mucosal damage, crypt loss, immune cell infiltration, and ↓ proinflammatory cytokines (IL-1, IL-6, IFN-γ and TNF-α)	TX527 suggests therapeutic potential for IBD management
D’Ambrosio et al. (1998) [37]	Cotransfected monocytic RAW264.7 cells with p40 promotor/reporter constructs and expression vectors of VDR and RXR-α	Expression of NF-κB pathway and cytokines following 1,25-OHD	1,25-OH D caused downregulation of NF-κB activation and ↓ in downstream IL-12	1,25-OHD may downregulate IL-12 production via downregulation of NF-kB activation and p40-kB sequence binding
Du et al. (2015) [38]	Cell study (HCT116, Caco-2, SW480) w/ TNF-α ± 1,25-OHD treatment; C57BL/6 mice w/ paricalcitol (vitamin D analog) treatment; Human colonic mucosal biopsies	Histology/immunostaining; Western blotting, RT-PCR, chromatin immunoprecipitation assay, myeloid perioxidase assay	TNF-α ↓ cells’ transepithelial electrical resistance (TER); > 70% VDR ↓ unable to counteract TNF-α activity and maintain TER; 1,25-OHD counteracts NF-κB by suppressing the myosin light chain kinase signaling pathway (MLCK); MLCK pathway activation = ↓ VDR in human biopsies; paricalcitol inhibits MLCK activation, regulates mucosal barrier, and ameliorates colitis in the mouse model	VDR is required to maintain and protect intestinal epithelial barrier
Meeker et al. (2014) [8]	*Smad3*^−/−^ mice fed w/ maintenance diet, high vitamin D diet (5 IU/g), or vitamin D-deficient (1 IU/g) diet infected with ~2 × 10^7^ CFU *Hb* in Brucella broth or Brucella broth alone (control)	*Helicobacter bilis* infection; Fecal polymerase chain reaction; serum vitamin D and calcium; histopathology; immunochemistry; epithelial and lamina propria leukocyte cell samples; Western blotting	↑ vitamin D diet after *H. bilis* infection = ↑ serum vitamin D w/o affecting calcium levels. ↓ dysplasia, ↓ inflammation (MAPK, NF-κB), ↓ IBD scores	Vitamin D intake suggests reduced colitis symptoms and delay progression to early stage carcinogenesis
Knackstedt et al. (2013) [13]	Female C57BL/6J mice fed normal chow or vitamin D-deficient diet; Acute colitis: 10 mg/kg AOM + 2% DSS treatment Colitis-associated cancer (CAC): 12 mg/kg AOM + 4% DSS treatment	Vitamin D quantification; colitis scoring; immunohistochemistry; protein extraction; immunoblotting; RT-PCR	AOM/DSS-induced acute colitis mice had ↓ body weight, ↑ blood loss, shortened colon, ↓ serum vitamin D compared to control; CAC model had mortality rate between 10–40%, shortened colon, ↓ serum vitamin D, TNF-α upregulated, RXRα and VDR downregulated	Vitamin D-deficient diet leads to increased colitis scores in the mouse model, downregulated RXRα, VDR, and decreased serum vitamin D levels

## Abbreviations

25-OHD	25-hydroxy vitamin D
1,25-OHD	1,25-duhydroxyvitamin D
AOM	azoxymethane
BMI	body mass index
CDC	Centers for Disease Control and Prevention
CDI	Clostridium difficile infection
COX-2	cyclooxygenase-2
CRP	C-reactive protein
DRI	Dietary Reference Intake
DSS	dextran sulfate sodium
ERK	extracellular signal related kinase
ESR	erythrocyte sedimentation rate
hTERT	Telomerase reverse transcriptase
IBD	inflammatory bowel disease
IL-6	interleukin-6
IU	international units
MLCK	myosin light chain kinase pathway
LL37	cathelicidin
MAPK	mitogen-activated protein kinase
PUMA	p53-upregulated modulator of apoptosis
RXRα	Retinoid X Receptor alpha
TNBS	2,4,6-trinitrobenzene sulfonic acid
TNF-α	tumor necrosis factor-alpha
UC	ulcerative colitis
USDHHS	United States Department of Health and Human Services
VDR	vitamin D receptor
